# Disease-Group-Specific Antimicrobial Use Patterns and Farm-Level Stewardship Features in Large-Scale Hungarian Swine Herds: A Multi-Farm Survey

**DOI:** 10.3390/ani16101570

**Published:** 2026-05-21

**Authors:** Ádám Kerek, László Gombos, Marietta Máté, László Ózsvári

**Affiliations:** 1 Department of Pharmacology and Toxicology, University of Veterinary Medicine Budapest, H-1078 Budapest, Hungary; 2 National Laboratory for Infectious Animal Diseases, Antimicrobial Resistance, Veterinary Public Health and Food Chain Safety, University of Veterinary Medicine Budapest, H-1078 Budapest, Hungary; mate.marietta@univet.hu (M.M.); ozsvari.laszlo@univet.hu (L.Ó.); 3 Wittmann Antal Multidisciplinary Doctoral School of Plant, Animal and Food Sciences, Széchenyi István University, H-9246 Mosonmagyaróvár, Hungary; drgomboslaci@gmail.com; 4 Agrofeed Research and Development Division, 10 Dunakapu Square, H-9022 Győr, Hungary; 5 Department of Veterinary Forensics and Economics, University of Veterinary Medicine Budapest, H-1078 Budapest, Hungary

**Keywords:** swine, antimicrobial use, antimicrobial stewardship, disease-group-specific treatment, *Streptococcus suis*, PRDC, pig production, vaccination, farm management, One Health, questionnaire survey

## Abstract

**Background**: Farm-level antimicrobial stewardship in swine production requires indication-specific knowledge of treatment patterns and the herd-level features associated with them. **Methods**: We analyzed questionnaire-based data collected in 2015 from 13 Hungarian swine farms covering 15,725 sows and their progeny. The survey captured production indicators, pathogen occurrence, vaccination, resistance-testing practices, drug costs, and disease-group-specific antimicrobial use. As a separate, non-mergeable descriptive temporal comparator, we also considered independent digital farm-monitoring data from three large-scale swine herds from 2022 to 2024. **Results**: The most frequently reported pathogens were *Mycoplasma hyopneumoniae* (13/13 farms), *Lawsonia intracellularis* (12/13), *Escherichia coli* (12/13), swine influenza virus (11/13), and *Streptococcus suis* (10/13). *S. suis* ranked as the leading damaging pathogen on 69% of farms. Among farms with antibiotic cost data (9/13), antibiotics accounted for a mean of 31.8% of veterinary drug expenditures. Among farms with treatment-by-indication data (8/13), the highest relative frequency of reported treatment events was linked to porcine respiratory disease complex, where doxycycline represented 38% of reported PRDC treatment events. Colistin dominated *E. coli*-associated diarrhea control, whereas beta-lactams were central for *S. suis*-related disease. In the 2022–2024 comparator dataset, enteric and respiratory disorders and arthritis remained the main recorded health problems, but corrected antimicrobial use was markedly lower in the later dataset. **Conclusions**: Antimicrobial use showed clear disease-group-specific patterns, supporting syndrome-focused stewardship rather than generic reduction targets.

## Simple Summary

Reducing antimicrobial use in pig production is now a major animal- and public-health goal, but meaningful reduction requires understanding why treatments are used at farm level. In this study, we analyzed questionnaire-based data from 13 large-scale Hungarian swine farms to identify the diseases, pathogens, management features, and preventive practices most closely linked to antibiotic use. Respiratory and enteric disease complexes dominated the health profile of the surveyed farms. The most frequently reported pathogens were *Mycoplasma hyopneumoniae*, *Lawsonia intracellularis*, *Escherichia coli*, swine influenza virus, and *Streptococcus suis*. Among farms with available treatment-by-indication data, the highest relative frequency of reported treatment events was linked to porcine respiratory disease complex, whereas colistin-based group medication was common for *E. coli*-associated diarrhea and beta-lactams were central for *S. suis*-related disease. Vaccination against *E. coli* in sows and against *M. hyopneumoniae* and PCV-2 in offspring was widespread among farms with available records, but diagnostic and resistance-testing intensity remained uneven. Antibiotics also represented a substantial share of veterinary drug costs. Findings from three additional independently monitored large-scale swine herds indicated that the same major health problems remained important, although antimicrobial use was lower in that separate comparator material. Overall, the findings support disease-group-specific, diagnostics-driven antimicrobial stewardship integrated with vaccination, biosecurity, and management.

## 1. Introduction

Antimicrobial use in pig production sits at the center of the One Health antimicrobial resistance debate because swine systems combine high animal density, repeated pathogen exposure, and strong pressure to maintain health and productivity under continuous commercial constraints [1,2,3]. At the policy level, reduction targets are often framed through national sales data or broad stewardship principles, yet such metrics cannot explain which disease syndromes, age groups, or herd-level decision patterns actually sustain antimicrobial demand on farms [4,5,6]. This limitation is not merely conceptual. Indication-based analyses in food-producing animals have shown that, in pigs, highest-priority critically important antimicrobials are used mainly for enteric and respiratory infections [7], while dose-based studies indicate that respiratory and enteric disease episodes are managed differently in the field, including differences in dosing accuracy and practical exposure patterns [8]. This gap is not trivial: the same total quantity of antimicrobial use may reflect very different epidemiological realities depending on whether treatments are driven by recurrent respiratory disease, post-weaning enteric disorders, *Streptococcus suis*-associated systemic disease, or poorly defined preventive routines. For stewardship interpretation, the reason for treatment is often more informative than the amount of drug alone [4,8].

For that reason, farm-level, indication-specific data remain essential. Disease ecology strongly shapes therapeutic behavior in swine practice. Porcine respiratory disease complex can involve interacting viral, bacterial, environmental, and management factors, often leading to repeated metaphylactic or group treatment, especially in growing and finishing pigs [9,10]. Recent work has reinforced that PRDC is better understood as a biologically layered syndrome than as a single-pathogen entity, with coinfections, pathogen diversity, and respiratory microbial community structure all influencing clinical expression and treatment pressure [11,12]. Enteric syndromes, particularly those linked to *Escherichia coli* and *Lawsonia intracellularis*, may also drive substantial oral medication pressure in suckling, nursery, and finishing phases, while *S. suis* occupies a special position as a highly consequential secondary pathogen associated with meningitis, septicemia, pneumonia, and arthritis [10,13]. This remains highly relevant in the post-weaning window, where diarrheal disease is still one of the clearest antibiotic-intensive bottlenecks in pig production; recent reviews and field studies continue to show that post-weaning diarrhea is linked to high antimicrobial consumption, often before susceptibility testing is available, while *L. intracellularis* remains a major ecological component of treatment-intensive enteric disease [14,15,16,17]. This pressure has also intensified interest in experimental modelling of porcine enteropathies and in antibiotic-alternative approaches aimed at reducing treatment dependence in pig production [18,19]. *S. suis* also warrants separate emphasis because it remains a major driver of antimicrobial use in weaned pigs, and its clinical impact may be intensified by viral and bacterial co-infections [20,21]. In practice, these syndromes differ not only in pathogenesis but also in route of administration, antimicrobial spectrum, duration of exposure, and the balance between individual versus group treatment.

Stewardship therefore depends on more than simply using fewer antibiotics. It depends on understanding the herd-level features and conditions associated with reported antimicrobial use, including those that make treatment necessary or, in some settings, habitual. Biosecurity, vaccination, housing design, all-in/all-out discipline, pathogen monitoring, and resistance testing can all modify treatment intensity and antimicrobial selection, but their farm-level implementation is often uneven [2,5,22]. Equally important, the effects of preventive tools are not uniform across production systems. Register-based studies from Danish pig production indicate that vaccination does not necessarily lead to reduced antimicrobial use. The relationships between vaccination, antimicrobial consumption, and productivity may be weak, nonexistent, or context dependent, varying with the targeted pathogen, herd type, and the structure of data recording [23,24,25]. Conversely, other field data have demonstrated reduced group medication after vaccination against *L. intracellularis*, underscoring that preventive success is pathogen- and system-specific rather than universal [26]. Earlier Hungarian work has already shown that animal-health management, technology level, and production performance are tightly interlinked, and that drug costs can account for a major share of veterinary expenditure on large-scale farms [9,22]. What remains less clearly described is how these herd-level conditions translate into concrete, disease-group-specific antimicrobial use patterns across commercial swine systems, particularly in datasets that preserve the original therapeutic logic of farm veterinarians.

This question is particularly relevant in historical farm datasets collected before the current European reporting architecture became more formalized, because such datasets capture decision behavior in a setting where treatment logic was recorded directly by practitioners and farm managers rather than reconstructed from centralized databases. These data are methodologically imperfect, but they are unusually informative: they reveal which pathogens were considered dominant, which syndromes repeatedly triggered treatment, which preventive programs were already in place, and where diagnostics and susceptibility testing did—or did not—appear to shape therapeutic choices [3,27]. That perspective remains useful today. Objective pathogen-monitoring studies in nursery and finisher pigs have shown that repeated laboratory surveillance can materially improve interpretation of coughing and diarrheal signals at unit level, reinforcing the value of disease-oriented herd data over purely aggregated consumption figures [28]. In the same vein, recent work on enteric disease ecology continues to show that treatment-intensive syndromes in growing pigs emerge from interactions between pathogens, management, and prevention, rather than from antimicrobial choice alone [14,17].

The aim of the present study was therefore to characterize disease-group-specific antimicrobial use patterns and farm-level stewardship features in a multi-farm survey of large-scale Hungarian swine herds. Using questionnaire-based data from 13 farms, we integrated production parameters, pathogen occurrence, vaccination records, resistance-testing information, veterinary drug costs, and treatment-by-indication summaries. Rather than estimating national antimicrobial consumption, our objective was to identify the herd-level structures that sustained antimicrobial demand and to translate them into a stewardship-oriented framework for large-scale swine production. To place this historical stewardship landscape in temporal context, we additionally considered summary-level 2022–2024 farm-monitoring data as a separate, non-mergeable comparator.

## 2. Materials and Methods

### 2.1. Study Design and Participating Farms

This study was designed as a cross-sectional, questionnaire-based, multi-farm observational survey of disease-group-specific antimicrobial use patterns and stewardship-relevant farm features in Hungarian commercial swine production. The source dataset was collected in 2015 using a structured questionnaire developed for veterinary, herd-health, and management assessment at farm level. Farms were included by convenience sampling, based on accessibility and willingness to provide herd-level information considered reliable and documentable by the responding farm manager or veterinarian. The questionnaire was administered either during on-farm professional consultations or, when adequate prior knowledge of the herd was already available, through direct communication with farm managers or attending veterinarians. Only data that respondents considered verifiable from existing farm records, annual summaries, laboratory reports, vaccination logs, treatment logs, or routine veterinary documentation were retained for analysis. Items based solely on unsupported recall or judged by the respondent to be unverifiable were excluded. A blank English version of the questionnaire is provided as Supplementary File S1. Different sections of the questionnaire were completed by the respondent most directly responsible for the respective domain: herd-structure, biosecurity, and technology sections by the farm manager/owner; disease, diagnostic, and treatment sections by the attending veterinarian; and expenditure-related sections by the farm financial manager and/or veterinarian, depending on local record-keeping practice. Because the source material was archival, a complete recruitment log was not preserved; therefore, the exact number of farms approached and the declining participation number could not be reconstructed retrospectively.

A total of 13 large-scale commercial swine farms from nine Hungarian counties were included, representing 15,725 sows and their progeny. For the purpose of this study, “large-scale” denoted commercially intensive holdings with breeding-herd inventories ranging from 575 to 2667 sows, or an equivalent finishing-only unit, operating under organized veterinary and production management conditions. Twelve holdings operated as mixed farrow-to-finish systems, whereas one holding was a finishing-only unit. For anonymity, farms were coded by letters (A–M). The questionnaire covered five major domains: production and herd-structure indicators; external and internal biosecurity and technology characteristics; pathogen occurrence, clinical problems, and herd-health programs; vaccination and resistance-testing practices; and veterinary drug expenditure and disease-group-specific antimicrobial use.

### 2.2. Survey Domains and Data Capture

Production variables included sow inventory, farrowing frequency, born-alive piglets per litter, weaning output, weaning age and weight, slaughter weight, daily gain, and mortality. Pathogen occurrence was recorded from farm-level, laboratory-confirmed diagnostic information available at the participating holdings. Pathogen occurrence reflected herd status considered relevant at the time of questionnaire completion based on current or prior laboratory-confirmed diagnoses known to the farm; it was not restricted to newly detected active infections occurring strictly within calendar year 2015. The questionnaire captured only whether pathogen presence had been confirmed by routine farm-level laboratory diagnostics; assay-specific methods (e.g., bacteriology, PCR, serology, or post-mortem confirmation) were not recorded in a harmonized manner and therefore could not be stratified retrospectively. In the questionnaire, pathogens were separated into two practical categories: pathogens considered part of the herd disease background and facultative or secondary pathogens causing substantial field damage under routine production conditions. Farms were also asked to rank the pathogens or disease complexes considered most important in everyday herd-health management.

Vaccination records captured preventive vaccination by target pathogen and production group. Resistance-testing information was collected from the most recent farm-level susceptibility results available to the participating veterinarians at the time of data capture. Because these results originated from routine diagnostic practice and were not generated within a harmonized, centrally standardized laboratory framework, they were summarized qualitatively at pathogen level in the present manuscript and used only for stewardship-oriented interpretation rather than formal cross-farm quantitative comparison. Production, vaccination, cost, and treatment summaries referred primarily to calendar year 2015, whereas susceptibility information referred to the most recent available farm-level laboratory results at the time of questionnaire completion.

Antimicrobial-use information was captured in two complementary ways. First, respondents described the general disease burden and the principal pathogens perceived to shape treatment demand on the farm. Second, eight farms provided disease-group-specific treatment information, including the reported active substance, production phase, route of administration, and treatment context. The analyzed disease groups were postpartum dysgalactia syndrome; *E. coli*-associated diarrhea; *S. suis*-related disease; porcine respiratory disease complex; joint and musculoskeletal disease; staphylococcal skin lesions; ileitis; enzootic pneumonia; general diarrhea; swine dysentery; prophylaxis associated with invasive procedures; and general preventive treatments. Because the historical questionnaire recorded reported treatment occurrence patterns rather than standardized dose-based metrics, these summaries were analyzed as disease- and production-phase-specific reported use events rather than as defined daily doses, treatment incidence, or biomass-adjusted exposure measures. For analytical purposes, one reported treatment event was defined as a unique farm × disease group × active substance × production phase × route-of-administration combination recorded in the questionnaire summaries. These events reflect the presence of a reported treatment practice, not its frequency, duration, or quantitative intensity.

### 2.3. Analytical Approach

Data analysis was primarily descriptive and stewardship-oriented. Farm-level pathogen prevalence was expressed as the proportion of farms reporting a given pathogen. Vaccination coverage was summarized among farms with available vaccination records, and antibiotic expenditure was expressed as the proportion of total veterinary drug costs among farms providing usable cost data. For disease-group-specific treatment visualization, individual active substances were aggregated into pharmacological classes when this improved interpretability and reduced excessive fragmentation of the historical treatment data.

All summaries were based on available-case analysis; therefore, denominators varied across analytical domains according to data completeness within the original questionnaire dataset (Table 1). Because the study was exploratory in design, included only 13 farms, and relied on partially incomplete domain-specific records, no inferential statistical modeling was performed. In addition, the original questionnaire was not designed to capture administered dose, treatment duration, numbers of treated animals, or bodyweight denominators in a standardized format; therefore, standardized quantitative antimicrobial-use indicators, treatment incidence estimates, and cross-farm exposure models could not be reconstructed retrospectively in a methodologically valid manner. The analytical objective was instead to identify and describe the main herd-level structures, disease syndromes, and therapeutic patterns that appeared to sustain antimicrobial demand across the surveyed farms.

### 2.4. Temporal Contextual Comparison with 2022–2024 Farm-Monitoring Data

To provide cautious temporal context, we additionally incorporated summary-level antimicrobial-use indicators from an independent, separately analyzed farm-monitoring dataset collected between January 2022 and June 2024 in three large-scale swine herds. The comparator dataset was derived from a digital herd-health recording system and summarized antimicrobial use by total mass (mg), corrected mg/PCU, calculated using the actual number of animals in the respective production groups, AMEG category, route of administration, treatment purpose, and age group. The three comparator farms were not part of the 2015 questionnaire survey, and the comparator material was not merged with the historical dataset at any stage of the analysis. AMEG categories were interpreted according to the European Medicines Agency classification, in which category B denotes “Restrict”, category C “Caution”, and category D “Prudence” [3].

These data were not pooled with the 2015 survey dataset and were not used for joint inferential or longitudinal analysis, because the two materials differed in farm composition, data-capture method, stewardship metrics, and analytical granularity. Instead, the 2022–2024 material was used exclusively as an external, summary-level temporal comparator to examine whether the syndrome-driven stewardship architecture reconstructed from the 2015 survey remained recognizable in a later, digitally recorded farm-monitoring environment operating under more formalized antimicrobial-governance conditions. Accordingly, all comparisons between the two datasets were interpreted descriptively and contextually, and no formal time-trend inference was attempted across the two study materials.

## 3. Results

### 3.1. Survey Coverage and Herd Production Context

The survey included 13 large-scale swine farms from nine Hungarian counties, representing 15,725 sows and their progeny. Twelve holdings operated as farrow-to-finish systems, whereas one was a finishing-only unit. The participating farms represented commercially intensive production systems. Across farms, the mean number of born-alive piglets per litter was 12.8, the mean annual farrowing frequency was 2.3, and the calculated output reached 29.6 piglets per sow per year. The mean number of weaned piglets was 26.33 per sow per year.

Despite this generally high-output production profile, inter-farm heterogeneity was substantial. Weaning age exceeded 28 days on five farms and ranged from 24.5 to 27 days on the others. Among farms with a nominal weaning age of 28 days, weaning weight ranged from 7.1 to 8.2 kg. Daily gain from weaning to slaughter exceeded 600 g/day on all evaluable farms but still ranged from 634 to 776 g/day. Mortality also varied markedly: in five of twelve farms with evaluable suckling-pig data, losses exceeded 1.5 piglets per litter, and annual deaths normalized to sow inventory ranged from 2.4 to 6.6 pigs per sow.

### 3.2. Farm-Level Pathogen Burden and Disease Hierarchy

**Figure 1:**
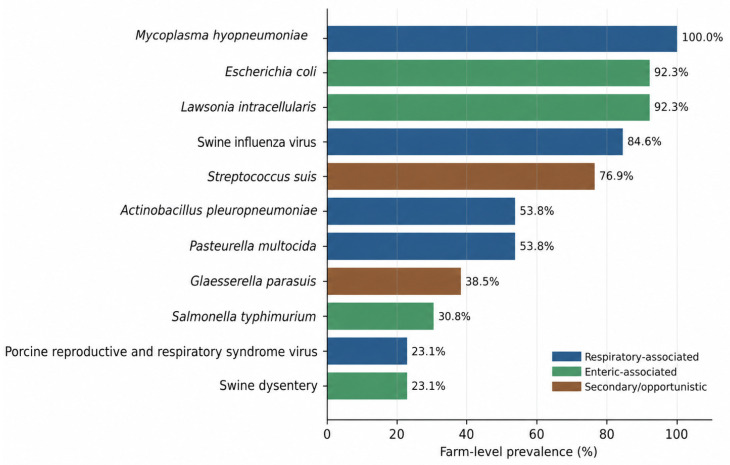
Farm-level prevalence of major pathogens in the 2015 Hungarian swine survey. Horizontal bars show the proportion of farms reporting each pathogen. Colors group pathogens into respiratory-associated, enteric-associated, secondary or opportunistic, and mixed/other categories. Farm-level prevalence was calculated from 13 farms.

The surveyed farms showed a broad pathogen burden, with respiratory, enteric, and secondary or opportunistic agents coexisting across most systems (Table 2; Figure 1). Among herd-background pathogens, *Mycoplasma hyopneumoniae* was reported on all 13 farms, *L. intracellularis* on 12 farms, and swine influenza virus on 11 farms. *Actinobacillus pleuropneumoniae* and *Pasteurella multocida* were each reported on seven farms, whereas porcine reproductive and respiratory syndrome (PRRS) virus and *Brachyspira hyodysenteriae* were each reported on three farms. Among facultative or secondary pathogens causing practical damage, *E. coli* was reported on 12 farms and *S. suis* on 10 farms, followed by *Glaesserella parasuis* on five farms and *Salmonella typhimurium* on four farms.

The hierarchy of practical damage was more informative than prevalence alone. *S. suis* emerged as the leading damaging pathogen on 69% of farms and appeared in the top three on 11 of 13 farms, highlighting its central role in therapeutic decision-making despite its classification as a secondary pathogen. Five farms reported more than three major damaging pathogens or disease complexes, indicating substantial syndromic complexity. This was consistent with the clinical pattern observed across production groups, in which respiratory disease, diarrhea, arthritis, and lameness recurred repeatedly in nursery and finishing units, alongside additional reproductive and neonatal problems in breeding and farrowing units.

### 3.3. Vaccination and Diagnostic Context

**Figure 2:**
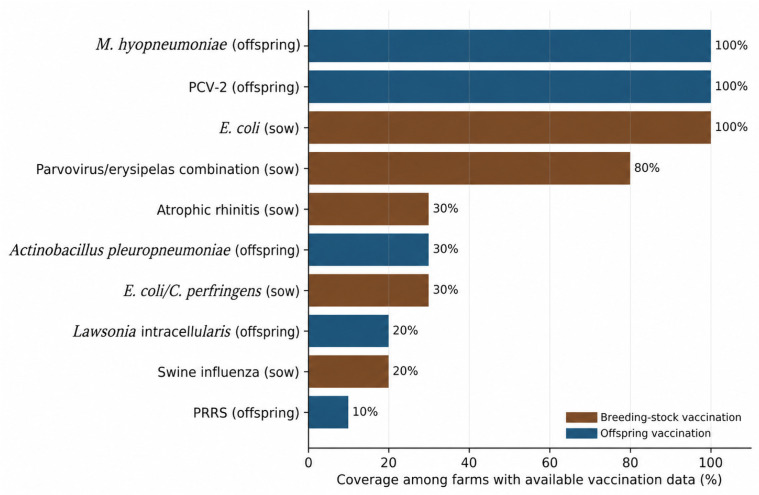
Vaccination coverage against key pathogens among reporting farms. Coverage is expressed among the 10 farms that provided usable vaccination data. Brown bars indicate breeding-stock vaccination; blue bars indicate offspring vaccination. PCV-2: Porcine circovirus type 2.

Preventive programs were common but unevenly distributed across farms. Vaccination records were available from 10 farms. Among these, all breeding herds were vaccinated against *E. coli*, and three also used a combined *E. coli* and *Clostridium perfringens* product. Vaccination against *M. hyopneumoniae* and porcine circovirus type 2 (PCV-2) in offspring was universal among farms with available records. By contrast, only two farms vaccinated breeding stock against swine influenza, three against atrophic rhinitis, three vaccinated offspring against *Actinobacillus pleuropneumoniae*, two against *L. intracellularis*, and one against porcine reproductive and respiratory syndrome (Figure 2; Table 3).

Resistance testing and pathogen identification were part of the diagnostic background but were not standardized across farms. The most frequently reported recent test results involved *S. suis*, *E. coli*, and *Pasteurella multocida*, with additional testing reported for *Staphylococcus* spp., *Salmonella* spp., *G. parasuis*, *Trueperella pyogenes*, and *A. pleuropneumoniae*. Qualitative susceptibility summaries indicated favorable reported susceptibility of *S. suis* to beta-lactams and of *E. coli* to colistin, gentamicin, and neomycin, whereas tetracyclines appeared less reliable for *S. suis* and, in several farms, also for *P. multocida* and *A. pleuropneumoniae* (Table 4). These summaries should be interpreted strictly as respondent-reported, farm-specific impressions of the latest available laboratory findings rather than as harmonized cross-farm susceptibility measurements.

### 3.4. Antibiotic Expenditure Within Veterinary Drug Costs

**Figure 3:**
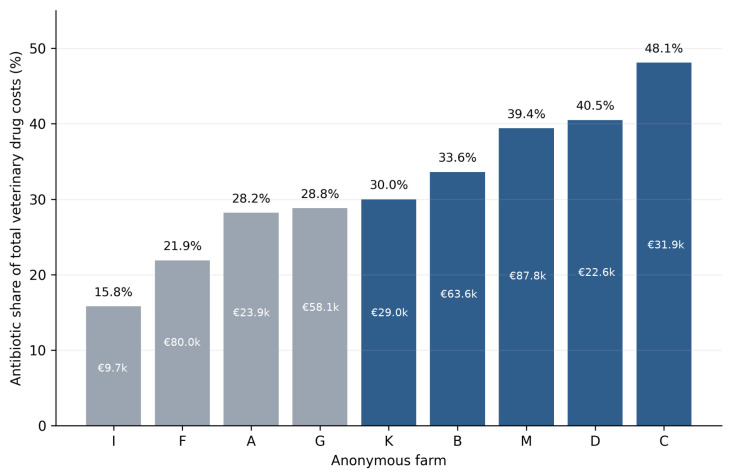
Antibiotic expenditure as a share of total veterinary drug costs among farms with available cost data. Bars show the share of antibiotic expenditure within total veterinary drug costs for the nine farms providing usable cost data. Internal labels indicate annual antibiotic expenditure in thousands of euros (1 EUR = 309.6 HUF). Farms are anonymized and sorted from the lowest to the highest antibiotic cost share.

Nine farms provided usable veterinary drug cost data. Across these farms, antibiotics accounted for a mean of 31.8% of total veterinary drug expenditure, ranging from 15.8% to 48.1% (Figure 3). The lowest antibiotic cost share was recorded on Farm I (15.8%), whereas Farms C, D, and M each exceeded 39%. These data show that antibiotics were a major, but highly variable, component of veterinary drug expenditure across the surveyed farms.

### 3.5. Disease-Group-Specific Antimicrobial Use Patterns

Eight farms provided disease-group-specific treatment information. Reported treatment events were distributed unevenly across syndromes, with the highest relative frequency linked to porcine respiratory disease complex (PRDC). Within this category, doxycycline accounted for 38% of reported treatments and was used predominantly via drinking water in nursery and finishing units. Sulfonamides, macrolides, chlortetracycline, florfenicol, amoxicillin, and injectable fluoroquinolones were also reported in specific farm contexts.

*E. coli*-associated diarrhea showed a distinct treatment profile dominated by colistin, particularly through drinking-water medication in nursery and finishing phases. In suckling pigs, injectable amoxicillin, enrofloxacin, gentamicin, and lincospectin-spectinomycin were also used. By contrast, *S. suis*-related disease was dominated by beta-lactams, especially amoxicillin, across breeding, nursery, and, less frequently, finishing phases. Florfenicol, ceftiofur, and ampicillin appeared as additional options. Joint and musculoskeletal syndromes also relied mainly on beta-lactams, with more selective use of oxytetracycline, ceftiofur, and penicillin-streptomycin.

**Figure 4:**
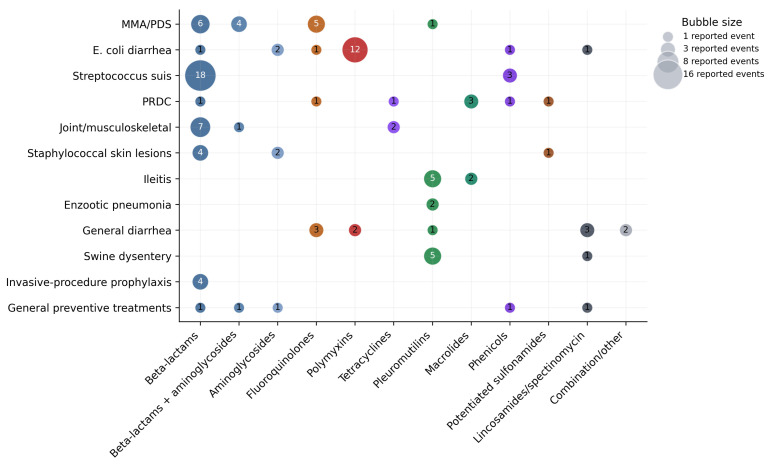
Disease-group-specific distribution of reported treatment events in the survey. Bubble size reflects the number of reported disease- and phase-specific treatment events aggregated by antimicrobial class from the narrative survey summaries. These values represent reported treatment occurrence patterns rather than biomass-adjusted exposure or standardized dose metrics. MMA/PDS: Mastitis, metritis, agalactiae/Postpartum dysgalactia syndrome. PRDC: Porcine Respiratory Disease Complex.

Enteric and intracellular syndromes displayed a third therapeutic pattern. Ileitis and swine dysentery were principally associated with pleuromutilins, especially tiamulin, with additional use of tylosin, doxycycline, or lincomycin in some farms. Staphylococcal skin lesions were treated mainly parenterally, most often with ceftiofur or gentamicin, although one farm reported herd-level sulfonamide treatment in fattening pigs. General diarrheal syndromes without a confirmed pathogen showed the broadest therapeutic dispersion, including enrofloxacin, colistin, tiamulin, aminoglycoside-containing combinations, and lincospectin-spectinomycin. Prophylaxis associated with invasive procedures and non-specific general preventive treatments were comparatively limited and were largely dominated by beta-lactams (Table 5; Figure 4).

### 3.6. Comparative Temporal Perspective

The independent 2022–2024 farm-monitoring dataset showed that enteric and respiratory disorders, together with arthritis, remained the most frequently recorded animal-health problems, corresponding to the major treatment-intensive syndromes identified in the 2015 survey. However, bodyweight-adjusted antimicrobial exposure was markedly lower in the later dataset. Total corrected antimicrobial use reached 405.17 corrected mg/PCU in 2022, then declined to corrected 94.59 mg/PCU in 2023 and remained at corrected 97.36 mg/PCU in the first half of 2024. The steepest reduction was observed in weaners, in which corrected use declined from 341.70 to 52.82 and then to corrected 42.35 mg/PCU.

Despite this reduction, route-of-administration patterns remained broadly stable. Drinking-water medication accounted for 93.8% of reported treatments in 2022–2024, indicating continued dominance of group oral treatment. At the same time, treatment-purpose records in the later dataset showed a shift toward more targeted use: in 2023, 83.7% of treatments were classified as therapeutic and 16.3% as metaphylactic. AMEG composition also changed over time, with category D agents dominating in 2022 (95.9%), followed by a larger share of category C compounds in 2023 (53.8%) and the first half of 2024 (54.7%), while category B use peaked transiently in 2023 (10.6%) before declining to 0.5% in 2024. These temporal indicators were included for contextual comparison only and were not merged analytically with the 2015 survey dataset.

## 4. Discussion

The central finding of this study is that antimicrobial use in large-scale swine production followed clear disease-group-specific patterns rather than non-specific treatment behavior. Respiratory and enteric disease complexes, together with *S. suis*-associated systemic and locomotor syndromes, constituted the dominant clinical architecture of treatment demand. From a stewardship perspective, this distinction is critical: antimicrobial pressure does not arise from an abstract background level of farm use, but from recurrent syndromic bottlenecks that differ in timing, route of administration, and preventive leverage points. In the present survey, porcine respiratory disease complex was the most treatment-intensive category, whereas *E. coli*-associated diarrhea, *S. suis*-related disease, ileitis, and swine dysentery showed distinct therapeutic profiles.

These signatures were biologically coherent. Respiratory disease in nursery and finishing units favored drinking-water medication and, in the surveyed farms, was closely linked to doxycycline-centered reported treatment-event patterns. By contrast, *S. suis*-related disease and musculoskeletal syndromes relied far more on beta-lactams and other injectable agents, reflecting the practical need for rapid systemic coverage in syndromes involving meningitis, septicemia, arthritis, or invasive secondary infection [10,13]. Enteric syndromes linked to *E. coli* were associated with polymyxin-driven group medication, whereas intracellular enteric disease such as ileitis favored pleuromutilins, which is consistent with the pharmacological logic described in swine practice texts [10]. In other words, the observed treatment structures were not accidental; they reflected the interaction between pathogen biology, production stage, and available administration routes.

The survey also shows why farm-level stewardship cannot be reduced to a single reduction target. Two farms may use antimicrobial treatment for entirely different reasons: one because of chronic respiratory instability in fatteners, another because of repeated neonatal and post-weaning enteric disease, and a third because of persistent *S. suis* pressure in nursery and breeding contexts. The practical implication is that effective stewardship must be syndrome-directed. For respiratory complexes, the highest leverage may come from pathogen monitoring, ventilation, all-in/all-out discipline, and selective vaccination. For enteric disease, leverage points may lie in farrowing hygiene, weaning management, feed transitions, and targeted preventive programs. For *S. suis*-dominated farms, attention to sow-to-piglet transmission, skin integrity, flooring, early arthritis signals, and rapid diagnostic confirmation may be decisive. This interpretation is consistent with European studies showing that antimicrobial use is linked not only to veterinary preference but also to biosecurity status, herd characteristics, and disease management structure [2,5,6].

Vaccination patterns in the present survey reinforce this view. Core vaccination against *E. coli* in breeding stock and against *M. hyopneumoniae* and PCV-2 in offspring was already deeply embedded among farms with available records, indicating that practitioners recognized the structural role of these pathogens in sustaining downstream treatment burden. By contrast, vaccination against *A. pleuropneumoniae*, *L. intracellularis*, swine influenza, atrophic rhinitis, and PRRS was much more selective, implying that these interventions were used primarily in herds with defined epidemiological need. This selective pattern is important: stewardship does not mean maximal vaccination against every pathogen, but rather precise deployment of preventive tools where herd-specific disease ecology justifies their use.

The resistance-testing summaries should be interpreted with caution, yet they add a clinically relevant dimension. Beta-lactam susceptibility for *S. suis* remained favorable in the reported farm results, whereas tetracyclines appeared less reliable for this pathogen. Likewise, the broad reported activity of colistin, gentamicin, and neomycin against *E. coli* in this historical dataset helps explain why those drugs occupied central places in field treatment logic at the time. At the same time, the historical prominence of colistin in these records must be interpreted in light of subsequent regulatory tightening and the current stewardship imperative to minimize polymyxin use in food-animal production. However, from a contemporary stewardship perspective, such findings do not justify complacency. They instead underscore the need to replace habitual compound selection with diagnostics-guided decision making and to re-evaluate historically successful regimens under current regulatory and resistance conditions [3,10,28].

The economic results further strengthen the stewardship argument. Antibiotics represented nearly one-third of veterinary drug costs on average and approached half of expenditure on some farms. This does not prove overuse; high expenditure may simply track high disease pressure. Yet it does show that antimicrobial demand is economically consequential enough that improvements in herd health management, vaccination strategy, and diagnostic precision are not only public-health goals but direct cost-control opportunities. Earlier Hungarian work similarly showed that drug costs, technology level, and production performance are tightly interconnected in swine operations [22]. The present survey extends that line of thinking by showing that the underlying drivers of cost are not merely “more disease” in general, but specific treatment-intensive disease groups.

This study has several limitations. First, it was based on questionnaire-derived farm data rather than prospectively standardized treatment records. In addition, because the study included only 13 farms enrolled by convenience sampling, the findings should not be interpreted as statistically representative of the broader Hungarian swine sector, but rather as analytically informative examples of farm-level therapeutic logic. Second, not every farm contributed usable data to every analytical domain, so denominators varied across production, vaccination, expenditure, and indication-specific treatment summaries, further limiting the basis for formal statistical comparison across farms. Third, susceptibility information was reported from the latest available farm results rather than from a harmonized, centrally re-tested panel. Fourth, the historical design does not allow causal attribution: we cannot conclude that a given vaccine program, biosecurity measure, or diagnostic habit directly reduced antimicrobial use. Accordingly, the patterns described in this study should be interpreted as descriptive and hypothesis-generating rather than causal. Finally, the study reflects a 2015 stewardship landscape; some compounds, decision rules, and regulatory boundaries have changed since then. Even with these constraints, the survey remains informative because it captures farm-level therapeutic logic in a way that highly aggregated national sales data cannot. In this sense, historical therapeutic logic remains directly relevant to contemporary stewardship, because it helps identify the recurrent syndromic and management bottlenecks that modern monitoring systems still need to address. A further limitation of the temporal comparison is that the 2022–2024 material originated from a separate, later monitoring system with different farm coverage and a different measurement architecture. Accordingly, the added time-trend perspective is interpretive rather than inferential.

The temporal comparison with the independent 2022–2024 Hungarian farm-monitoring dataset adds interpretive context. Enteric disease, respiratory disease, and arthritis remained the central treatment-intensive syndromes, indicating that the core syndromic architecture identified in 2015 persisted. What changed more clearly was the measurement environment and, with it, the structure of recorded antimicrobial deployment. The later period showed a sharp reduction in corrected mg/PCU, especially in weaners, alongside continued dominance of drinking-water medication and a stronger tilt toward recorded therapeutic rather than metaphylactic use. Because the datasets differ in farm composition, granularity, and capture method, this pattern cannot be read as a formal longitudinal effect. Nevertheless, the comparison is compatible with a more explicitly monitored and record-driven later stewardship setting, while the main treatment-intensive syndromes remained recognizable across the two periods.

## 5. Conclusions

In this multi-farm survey of large-scale Hungarian swine herds, antimicrobial use followed clear disease-group-specific patterns shaped by pathogen burden, production stage, route of administration, vaccination structure, and the uneven use of diagnostics. Porcine respiratory disease complex, *E. coli*-associated diarrhea, *S. suis*-related disease, ileitis, and swine dysentery emerged as the principal syndromic contributors to reported treatment demand, each with its own pharmacological signature. Antibiotic expenditure represented a substantial share of veterinary drug costs, confirming that stewardship is both a health and an economic issue. When interpreted alongside independent farm-monitoring data from 2022 to 2024, the 2015 survey is consistent with the view that the core treatment-intensive syndromes remained recognizable over time, even as corrected antimicrobial exposure declined and the recorded balance between metaphylactic and therapeutic use changed in the later monitoring period.

The practical message is straightforward: prudent antimicrobial stewardship in commercial pig production should target the major treatment-intensive syndromes that sustain group medication and repeated exposure, rather than relying on generic reduction rhetoric alone. Farm-specific integration of vaccination, pathogen monitoring, resistance testing, biosecurity, and production-stage-aware decision making is likely to provide a more credible basis for sustained antimicrobial reduction than generic reduction targets alone, while supporting herd health and production stability. This comparator should be interpreted cautiously, because the two datasets were generated using different study designs and recording architectures; however, it provides useful contextual support for the continued relevance of syndrome-focused stewardship.

## Figures and Tables

**Table 1:** Survey structure, study population, and data completeness.

Domain	Value	Notes
Participating farms	13	Anonymous farms coded A-M.
Geographic coverage	9 Hungarian counties	Hajdú-Bihar, Baranya, Bács-Kiskun, Komárom-Esztergom, Tolna, Győr-Moson-Sopron, Csongrád, Somogy, and Zala
Total sow inventory represented	15,725	Across participating farms.
Farm type	12 farrow-to-finish; 1 finishing-only	Farm E was a finishing-only unit.
Farms with herd-background pathogen data	13	Laboratory-confirmed pathogen occurrence.
Farms with secondary pathogen/damage data	13	Facultative or secondary pathogens causing practical losses.
Farms with vaccination records	10	No vaccination data were available for Farms D, E, and H.
Farms with antibiotic cost data	9	A, B, C, D, F, G, I, K, and M
Farms with disease-group-specific AMU data	8	C, D, E, F, G, I, L, and M

**Table 2:** Farm-level prevalence of major pathogens relevant to antimicrobial decision making.

Pathogen	Pathogen Type	Farms Positive	Prevalence (%)
*Mycoplasma hyopneumoniae*	Primary/respiratory	13/13	100.0
*Lawsonia intracellularis*	Primary/enteric	12/13	92.3
*Escherichia coli*	Secondary/enteric	12/13	92.3
Swine influenza virus	Primary/respiratory	11/13	84.6
*Streptococcus suis*	Secondary/systemic	10/13	76.9
*Actinobacillus pleuropneumoniae*	Primary/respiratory	7/13	53.8
*Pasteurella multocida*	Respiratory/opportunistic	7/13	53.8
*Glaesserella parasuis*	Secondary/respiratory/systemic	5/13	38.5
*Salmonella typhimurium*	Secondary/enteric	4/13	30.8
PRRS virus	Primary/respiratory/reproductive	3/13	23.1
*Brachyspira hyodysenteriae*	Primary/enteric	3/13	23.1

**Table 3:** Vaccination coverage against key pathogens among farms with available vaccination records. PCV-2: Porcine circovirus type 2. PRRS: Porcine reproductive and respiratory syndrome.

Vaccine Target	Target Group	Coverage	Coverage (%)	Reported Coverage Pattern
*Escherichia coli*	Sows	10/10	100.0	Reported in all farms with available vaccination records.
*Escherichia coli + Clostridium perfringens*	Sows	3/10	30.0	Reported on three farms.
Parvovirus + erysipelas	Sows	8/10	80.0	Reported on eight farms.
Swine influenza	Sows	2/10	20.0	Reported on two farms.
Atrophic rhinitis	Sows	3/10	30.0	Reported on three farms.
*Mycoplasma hyopneumoniae*	Offspring	10/10	100.0	Reported in all farms with available vaccination records.
PCV-2	Offspring	10/10	100.0	Reported in all farms with available vaccination records.
*Actinobacillus pleuropneumoniae*	Offspring	3/10	30.0	Reported on three farms.
*Lawsonia intracellularis*	Offspring	2/10	20.0	Reported on two farms.
PRRS	Offspring	1/10	10.0	Reported on one farm.

**Table 4:** Respondent-reported susceptibility information relevant to historical treatment selection in the survey.

Pathogen	Reported Options Perceived as Favorable	Reported Limitations	Historical Treatment Context
*Streptococcus suis*	Beta-lactams were reported as consistently favorable; florfenicol, tiamulin, tilmicosin, tulathromycin, and lincospectin-spectinomycin were also described as useful.	Tetracyclines were generally considered unreliable in the reported farm results.	Consistent with continued farm-level reliance on beta-lactam-centered regimens.
*Escherichia coli*	Colistin was reported as active on all farms in the historical dataset; gentamicin and neomycin also showed favorable activity; cephalosporins and fluoroquinolones performed well.	Amoxicillin, tetracyclines, and sulfonamides were described as poor options in many farm results.	Consistent with historical farm-level reliance on colistin, gentamicin, and neomycin.
*Pasteurella multocida*	Most tested compounds were considered useful; beta-lactams, florfenicol, and macrolides were described as practical options.	Reduced tetracycline reliability was reported on several farms.	Compatible with use of narrower respiratory treatment options when supported by local diagnostics.
*Actinobacillus pleuropneumoniae*	Beta-lactams, florfenicol, tiamulin, and macrolides were reported as effective.	Tetracyclines were strongly resistant in the two farms with recent reported tests.	Consistent with limited utility of tetracyclines in farms with recent reported test results.
*Glaesserella parasuis*	Very good reported susceptibility to most tested agents.	Sulfonamides were the main exception.	Consistent with broad reported activity except for sulfonamides.

**Table 5:** Disease-group-specific antimicrobial use patterns in the surveyed farms (part I). PDS: postpartum dysgalactia syndrome. The historical questionnaire used the combined term MMA/PDS; in the present manuscript, postpartum dysgalactia syndrome is used as the preferred current term.

Disease Group	Principal Production Stage(s)	Predominant Route(s)	Leading Reported Agents	Observed Field-Use Pattern
Postpartum dysgalactia syndrome (historically recorded as MMA/PDS)	Breeding/farrowing	Mainly injection: occasional water or top-dress.	Enrofloxacin; penicillin-streptomycin; amoxicillin	Primarily individual or small-group therapeutic intervention.
*Escherichia coli*-associated diarrhea	Farrowing, nursery, finishing	Mostly drinking water; injections in suckling pigs.	Colistin; amoxicillin; gentamicin; enrofloxacin; lincospectin-spectinomycin	High reliance on group medication in post-weaning/finishing phases.
*Streptococcus suis*-related disease	Breeding, nursery, finishing	Injection, drinking water, and feed.	Amoxicillin; florfenicol; ceftiofur; ampicillin	Beta-lactam-centered treatment pattern across multiple stages.
PRDC	Nursery and finishing	Mostly drinking water; selected injections; occasional feed medication.	Doxycycline; sulfonamides; tilmicosin, tilvalosin; florfenicol; amoxicillin; marbofloxacin	Highest relative frequency of reported treatment events among the surveyed disease groups.
Joint and musculoskeletal disease	Farrowing, nursery, breeding gilts	Mainly injection.	Amoxicillin; oxytetracycline; ceftiofur; penicillin-streptomycin	Predominantly beta-lactam-based treatment pattern.
Staphylococcal skin lesions	Farrowing, nursery, finishing	Mostly injections; rare herd-level water medication.	Ceftiofur; gentamicin; sulfonamides	Usually individual treatment, except one herd-level finishing outbreak.
Ileitis	Finishing	Drinking water and feed.	Tiamulin; tylosin; doxycycline	Pleuromutilin-dominant enteric treatment pattern.
Enzootic pneumonia	Breeding and nursery	Treatment reported in one herd.	Tiamulin	Reported in one herd.
General diarrhea (pathogen not confirmed)	Farrowing, nursery, finishing	Injection and drinking water.	Enrofloxacin; colistin; tiamulin; amoxicillin-gentamicin; lincospectin-spectinomycin	Broad therapeutic dispersion across reported agents.
Swine dysentery	Breeding, nursery, finishing	Mainly drinking water; occasional feed.	Tiamulin; lincomycin	Predominantly pleuromutilin-based reported treatment pattern.
Invasive-procedure prophylaxis	Neonatal/farrowing	Injection.	Amoxicillin; ceftiofur	Low-volume prophylactic use around invasive procedures.
General preventive treatments	Breeding, neonatal, nursery, finishing	Mixed.	Amoxicillin; gentamicin; florfenicol; lincomycin; penicillin-streptomycin	Mixed non-specific reported preventive treatment pattern.

## Data Availability

The data presented in this study are available from the corresponding author on reasonable request.

## Supplementary Materials

The following supporting information can be downloaded at: https://www.mdpi.com/article/10.3390/ani16101570/s1, File S1: Questionnaire used in the study.

## Abbreviations

The following abbreviations are used in this manuscript:

AMR	antimicrobial resistance
AMU	antimicrobial use
APP	*Actinobacillus pleuropneumoniae*
PCV-2	porcine circovirus type 2
PDS	postpartum dysgalactia syndrome
PRDC	porcine respiratory disease complex
PRRS	Porcine reproductive and respiratory syndrome
