# Supplementary material for: Disease-Group-Specific Antimicrobial Use Patterns and Farm-Level Stewardship Features in Large-Scale Hungarian Swine Herds: A Multi-Farm Survey
Source: Animals (Basel). 2026 May 21;16(10):1570. doi: 10.3390/ani16101570 (PMC13203145; doi:10.3390/ani16101570)
Supplement: Supplementary file 1 [file animals-16-01570-s001.zip › animals-4304965-supplementary.pdf]

Farm Checklist

For the year 2015

I, the undersigned, declare that I have been duly informed about the purpose of this questionnaire and the planned scientific use of the data. I acknowledge that the data provided by me may be used for research and scientific publication purposes in anonymized, aggregated form. By completing this questionnaire, I voluntarily consent to the above-described use and processing of the data.

Name: ____________________

Position/Institution: ____________________

Date: ____________________

Signature: ____________________

Section A. Farm manager / owner questionnaire

Respondent: farm manager / owner

Instructions: circle or underline the applicable option(s).

County: ____________________

Pig density in the area: ______ large-scale farms within a 5 km radius

Basic technical data

Production phase(s): breeding only / finishing only / breeding and nursery / breeding, nursery, and finishing / includes a pre-finishing unit

Other: ____

Genetics and breeding data

• Breeding sows: ______ animals - when is an animal counted as a sow?: at insemination / after one farrowing

• Breeding boars: ______ animals; semen purchase: YES / NO

• Total number of inseminations: ______

• Replacement gilts introduced: ______

• Total number of farrowings: ______

• Average number of live-born piglets per litter: ______ piglets/sow

• Number of weaned piglets: ______ piglets/sow; weaning age: ______ days; average body weight: ______ kg/piglet

• Age at transfer to the finishing unit: ______ days; average body weight: ______ kg/animal

• Number of pigs marketed per year: ______; average age at marketing: ______ days; average body weight: ______ kg/animal

• Total live weight sold: ______ kg

• Total annual mortality: ______ animals, of which:

• suckling piglets: ______; sows: ______; nursery pigs: ______; pre-finishing and finishing pigs: ______

• Total annual cull sales: ______ animals, of which:

• sows: ______; nursery pigs: ______; pre-finishing and finishing pigs: ______

• Mortality rate - basis of calculation: placed / present / born

• Overall farm: ______%; suckling piglets: ______%; nursery: ______%; pre-finishing: ______%; finishing: ______%

External biosecurity

• Fence: YES / NO / N/A

• Hand and foot disinfection: YES / NO / N/A

• Wheel disinfection: YES / NO / N/A

• Vehicle traffic entering the farm from outside: YES / NO / N/A

• Carcass storage facility located on the fence line: YES / NO

• Animal loading ramp: YES / NO

• Cleaning frequency: after every transport / weekly / less frequently

• Quarantine building: YES / NO; if yes, distance from the farm: ______ km; separate staff: YES / NO

• Animal purchases: YES / NO; domestic / foreign; occasional / regular

Internal biosecurity

• Black-and-white changing room: YES / NO / N/A

• Work clothing: YES (provided by the farm) / NO (no change of clothes required) / N/A (employees change clothes, but bring their own)

• Footbath in front of pens/rooms: YES / NO / N/A

• Personnel separated by production unit: YES / NO / N/A (in principle yes, but mixing may occasionally occur)

• Water supply: mains / water tower / other: ____________________

• Annual frequency of laboratory sampling: ____________________

Technology

Feeding system and feed type by production unit:

Production unit	System	Feed form
Breeding stock	traditional / renovated / new	meal / pelleted / wet / moistened
Nursery	traditional / renovated / new	meal / pelleted / wet / moistened
Pre-finishing	traditional / renovated / new	meal / pelleted / wet / moistened
Finishing	traditional / renovated / new	meal / pelleted / wet / moistened

• Total feed use on the farm: ______ tonnes, of which:

• gestating sows: ______ tonnes (daily ration: ______ kg/day)

• lactating sows: ______ tonnes (daily ration: ______ kg/day)

• baby starter: ______ tonnes (for how many days: ______)

• prestarter: ______ tonnes (for how many days: ______)

• piglet feed (1-2-... combined): ______ tonnes (for how many days: ______)

• finisher feed (1-2-... combined): ______ tonnes (for how many days: ______)

• All in / all out: farrowing YES / NO / N/A; group sow housing YES / NO / N/A; nursery YES / NO / N/A; pre-finishing YES / NO / N/A; finishing YES / NO / N/A

• (N/A: there is a downtime period, but rooms are not emptied and repopulated simultaneously.)

• Flooring type: breeding unit / nursery / pre-finishing / finishing:

____

• Rodent control: YES / NO; if yes: no rodents / occasionally present / not effective

• Fly control: YES / NO; if yes: no flies / occasionally present / not effective

• Average daily weight gain (g/day): nursery ______; pre-finishing ______; finishing ______

Farm Checklist - Veterinarian Form

I, the undersigned, declare that I have been duly informed about the purpose of this questionnaire and the planned scientific use of the data. I acknowledge that the data provided by me may be used for research and scientific publication purposes in anonymized, aggregated form. By completing this questionnaire, I voluntarily consent to the above-described use and processing of the data.

Name: ____________________

Position/Institution: ____________________

Date: ____________________

Signature: ____________________

Respondent: veterinarian

Instructions: circle or underline the applicable option(s).

Laboratory-confirmed diseases / pathogens present on the farm

PRRS - APP - Mhyo - Pasteurella - atrophic rhinitis - dysentery - ileitis - SIV - PCV-2

Other infectious diseases: ____

Laboratory-confirmed facultative pathogens / secondary pathogens causing damage:
____

Please rank the diseases that most strongly determine the life of the farm and for which antibiotics are used:

1. __________________________
2. __________________________
3. __________________________
4. __________________________
5. __________________________
6. __________________________

Typical clinical signs associated with herd-health problems on the farm

breeding sow: high mortality / many sudden deaths / nervous signs / diarrhea (bloody / non-bloody) / constipation / epistaxis / bloody foamy nasal discharge / watery serous nasal discharge / conjunctivitis / salivation / vomiting / anemia / skin lesions / cyanosis of ears and limbs / scaling / jaundice / skin hemorrhages / rashes-blisters / swelling above joints / distorted snout / lameness >2% / coughing / sneezing / dyspnea / other: __________

replacement gilt: high mortality / many sudden deaths / nervous signs / diarrhea (bloody / non-bloody) / constipation / epistaxis / bloody foamy nasal discharge / watery serous nasal discharge / conjunctivitis / salivation / vomiting / anemia / skin lesions / cyanosis of ears and limbs / scaling / jaundice / skin hemorrhages / rashes-blisters / swelling above joints / distorted snout / lameness >2% / coughing / sneezing / dyspnea / other: __________

suckling piglet: high mortality / many sudden deaths / nervous signs / diarrhea (bloody / non-bloody) / constipation / epistaxis / bloody foamy nasal discharge / watery serous nasal discharge / conjunctivitis / salivation / vomiting / anemia / skin lesions / cyanosis of ears and limbs / scaling / jaundice / skin hemorrhages / rashes-blisters / swelling above joints / distorted snout / lameness >2% / coughing / sneezing / dyspnea / other: __________

nursery: high mortality / many sudden deaths / nervous signs / diarrhea (bloody / non-bloody) / constipation / epistaxis / bloody foamy nasal discharge / watery serous nasal discharge / conjunctivitis / salivation / vomiting / anemia / skin lesions / cyanosis of ears and limbs / scaling / jaundice / skin hemorrhages / rashes-blisters / swelling above joints / distorted snout / lameness >2% / coughing / sneezing / dyspnea / other: __________

pre-finishing: high mortality / many sudden deaths / nervous signs / diarrhea (bloody / non-bloody) / constipation / epistaxis / bloody foamy nasal discharge / watery serous nasal discharge / conjunctivitis / salivation / vomiting / anemia / skin lesions / cyanosis of ears and limbs / scaling / jaundice / skin hemorrhages / rashes-blisters / swelling above joints / distorted snout / lameness >2% / coughing / sneezing / dyspnea / other: __________

finishing: high mortality / many sudden deaths / nervous signs / diarrhea (bloody / non-bloody) / constipation / epistaxis / bloody foamy nasal discharge / watery serous nasal discharge / conjunctivitis / salivation / vomiting / anemia / skin lesions / cyanosis of ears and limbs / scaling / jaundice / skin hemorrhages / rashes-blisters / swelling above joints / distorted snout / lameness >2% / coughing / sneezing / dyspnea / other: __________

Abattoir inspection results

Abattoir findings: latest / averaged; available / not available

Affected organs: lung / liver / intestine / heart / kidney / other: __________

Lungs:

- proportion of pleuritis in the diaphragmatic lobes: ______ % / total SPES: ______
- proportion of pleuritis in the cranial lobes: ______ % / total
- lesions characteristic of M. hyopneumoniae: ______ % / total
- severity: severe / moderate / mild; affected area(s)
- liver lesions: ______ % / total

Treatments

• Anthelmintic treatment: YES / NO; active ingredient: __________; oral / injectable

• Treated groups: breeding stock / nursery / pre-finishing / finishing

• Fecal monitoring: YES / NO; frequency: __________; are clinical signs present? YES / NO

• Coccidiosis treatment: YES / NO

• Monitoring: YES / NO; frequency: __________; are clinical signs present? YES / NO

• External parasite control: YES / NO; active ingredient: __________

• Treated groups: breeding stock / nursery / pre-finishing / finishing

• Monitoring: YES / NO; frequency: __________; are clinical signs present? YES / NO

• ZnO use: YES / NO

• Treated groups: nursery / pre-finishing / finishing

• Feed name: __________ dosage: __________ mg/kg

• Feed name: __________ dosage: __________ mg/kg

• Iron injection: YES / NO; product name: __________

• Dosing frequency: __________ total amount: __________ mg/piglet

• Use of alternative medicinal products: YES / NO

• Treated groups: breeding stock / suckling piglets / nursery / pre-finishing / finishing

• Diseases/signs treated in this way: _______________________________________

Results of the latest resistance tests for the given pathogen

(Based on the latest examination. Please enter the active substances in the antibiotic columns.)

Pathogen	Susceptible antibiotics	Intermediate antibiotics	Resistant antibiotics

Farm Checklist - Farm manager and veterinarian (economic data)

I, the undersigned, declare that I have been duly informed about the purpose of this questionnaire and the planned scientific use of the data. I acknowledge that the data provided by me may be used for research and scientific publication purposes in anonymized, aggregated form. By completing this questionnaire, I voluntarily consent to the above-described use and processing of the data.

Name: ____________________

Position/Institution: ____________________

Date: ____________________

Signature: ____________________

Respondent: farm manager and veterinarian

Instructions: circle or underline the applicable option(s).

Animal-health costs (HUF/year)

• Total: __________ (direct purchases + feed-medicated products + veterinarian)

• Of this: vaccines __________; reproductive hormones __________

• Antibiotics: in-feed __________; outside feed __________

• Internal antiparasitics __________; external antiparasitics __________; coccidiostats __________; disinfectants __________; vitamins __________

• Total farm costs in the given year: __________ HUF

Vaccinations on the farm

Disease	Vaccine name	Number of doses used in 2015

Disinfection

Product used: ____________________ quantity used in 2015: ____________________

Product used: ____________________ quantity used in 2015: ____________________

Product used: ____________________ quantity used in 2015: ____________________

Product used: ____________________ quantity used in 2015: ____________________

Antibiotic use

(Please also include products incorporated into feed. In such cases, it is sufficient to indicate the dose in mg/bw kg or mg/kg feed.)

Active ingredient	Product	Age group	Annual amount used (units/L/kg/etc.)	Treated disease(s)/pathogen(s)
